# Cephalometric variables used to predict the success of interceptive treatment with rapid maxillary expansion and face mask. A longitudinal study

**DOI:** 10.1590/2176-9451.20.1.085-096.oar

**Published:** 2015

**Authors:** Daniele Nóbrega Nardoni, Danilo Furquim Siqueira, Mauricio de Almeida Cardoso, Leopoldino Capelozza

**Affiliations:** 1 Master's student in Oral Biology, Sacred Heart University (USC); 2 Professor, Undergraduate and postgraduate program in Orthodontics, USC

**Keywords:** Angle Class III malocclusion, Prognosis, Discriminant analysis

## Abstract

**INTRODUCTION:**

Prognosis is the main limitation of interceptive treatment of Class III malocclusions. The interceptive procedures of rapid maxillary expansion (RME) and face mask therapy performed in early mixed dentition are capable of achieving immediate overcorrection and maintenance of facial and occlusal morphology for a few years. Individuals presenting minimal acceptable faces at growth completion are potential candidates for compensatory orthodontic treatment, while those with facial involvement should be submitted to orthodontic decompensation for orthognathic surgery.

**OBJECTIVES:**

To investigate cephalometric variables that might predict the outcomes of orthopedic treatment with RME and face mask therapy (FM).

**METHODS:**

Cephalometric analysis of 26 Class III patients (mean age of 8 years and 4 months) was performed at treatment onset and after a mean period of 6 years and 10 months at pubertal growth completion, including a subjective facial analysis. Patients was divided into two groups: success group (21 individuals) and failure group (5 individuals). Discriminant analysis was applied to the cephalometric values at treatment onset. Two predictor variables were found by stepwise procedure.

**RESULTS:**

Orthopedic treatment of Class III malocclusion may have unfavorable prognosis at growth completion whenever initial cephalometric analysis reveals increased lower anterior facial height (LAFH) combined with reduced angle between the condylar axis and the mandibular plane (CondAx.MP).

**CONCLUSION:**

The results of treatment with RME and face mask therapy at growth completion in Class III patients could be predicted with a probability of 88.5%.

## Abstract (pt)

**INTRODUÇÃO:**

a principal limitação do tratamento interceptivo das más oclusões de Classe III está no prognóstico. Os procedimentos interceptivos de expansão rápida da maxila e de tração reversa, adotados ao início da dentição mista, são capazes de propiciar sobrecorreção imediata e manutenção da morfologia facial e oclusal por alguns anos. Pacientes que, ao final do crescimento, apresentam, no mínimo, faces aceitáveis, são candidatos ao tratamento ortodôntico compensatório, ao passo que aqueles com comprometimento facial deveriam ser submetidos a tratamento ortodôntico descompensatório para cirurgia ortognática.

**OBJETIVO:**

investigar variáveis cefalométricas preditoras dos resultados do tratamento ortopédico com expansão rápida da maxila e tração reversa (ERM e TM).

**MÉTODOS:**

uma avaliação cefalométrica foi aplicada, ao início do tratamento, em 26 crianças com má oclusão de Classe III (média de idade de 8 anos e 4 meses). Após um período médio de 6 anos e 10 meses, ao final do crescimento pubertário. sob o crivo de uma análise facial subjetiva, foram constituídos dois grupos, sendo um grupo de sucesso (21 pacientes) e um grupo de insucesso (5 pacientes). Análise discriminante foi aplicada aos valores cefalométricos ao início do tratamento, por meio do procedimento *stepwise*, assim, identificamos duas variáveis preditoras.

**RESULTADOS:**

o tratamento ortopédico de uma má oclusão Classe III pode ter prognóstico desfavorável ao final do crescimento quando, nos registros cefalométricos iniciais, for observada uma altura facial anteroinferior aumentada (AFAI) associada a uma diminuição do ângulo entre o eixo condilar e o plano mandibular (CondAx.PM).

**CONCLUSÃO:**

os resultados para o final de crescimento de um tratamento com ERM e TM, para cada novo paciente com má oclusão de Classe III, poderiam ser previstos com uma probabilidade de acerto de 88,5%.

## INTRODUCTION

Treatment of Class III malocclusions is particularly limited in its prognosis^1^^-^ 4 which is usually complicated in cases of skeletal malocclusion with genetic determination.^5^^,^ 6 Subjects with malocclusion resulting from sagittal plane imbalance between the maxilla and the mandible are referred to as Class III malocclusion patients. This pattern includes subjects with maxillary retrusion and/or mandibular prognathism,^7^^,^ 8 regardless of the molar relationship established between dental arches.^5^^,^ 6 ^,^ 7 ^,^ 9 ^,^ 10 Although malocclusion tends to present a Class III molar relationship, it does not always express association with the severity of skeletal relationship^6^ and, as a consequence, with facial balance. This process depends on growth pattern and raises uncertainty over the stability of results after the active period has finished. Such uncertainties go beyond occlusal relationships and may compromise facial balance.

Skeletal discrepancies may, therefore, not only lead to malocclusion, but also to disharmony capable of impacting facial balance in a negative way.^6^

In fact, a limited number of studies demonstrate that Class III patients, who are in permanent dentition and have reached total facial growth, present characteristics that could have been observed at an early age.^7^^,^ 11 ^,^ 12 ^,^ 13 Additionally, the morphogenetic patterns of each patient remains during growth.

One of the protocols that is considered as effective in the orthopedic treatment of Class III malocclusions consists of RME associated with face mask therapy,^14^^-^ 18 preferably initiated in early mixed dentition more than it is in late mixed dentition.^19^^-^ 23

Patients who have received orthopedic treatment are admittedly benefitted during the active phase of treatment with favorable results not only for greater maxillary growth, about four times greater (from 1.9 mm to 2.3 mm), but also for restriction of mandibular growth due to redirecting the condyle upward and forward (from 1.3 to 3 mm) when compared to patients who have not received any kind of treatment.^1^^,^ 19 ^,^ 21 ^,^ 22 ^,^ 24 In general, positive overjet may be obtained in most patients after 6 to 9 months of treatment.^24^ However, after a four-year follow-up, 25% of patients relapse into anterior crossbite or negative overjet. These data have been reported by Ngan et al^24^ and were obtained from patients who presented excessive mandibular horizontal growth partially compensated by incisors, were at puberty and, therefore, had an unsatisfactory prognosis at growth completion.

Thus, results generally capable of providing immediate overcorrection and maintenance of facial and occlusal morphology for a few years have a long-term prognosis that is absolutely dependable on facial growth pattern. Orthodontic success or identification of which patients would benefit from early orthopedic treatment performed to disguise skeletal discrepancy requires growth prediction.^25^ Battagel^26^ was one of the first investigators who recognized the need for developing a model of predictors that fulfill this purpose. With similar intentions, Baccetti et al^25^ and Ghiz, Ngan and Gunel^27^ conducted retrospective studies, selecting cephalometric variables capable of predicting the future growth of a Class III patient. Ghiz et al^27^ found that the cephalometric variables for the mandible (size, length and gonial angle) were related to unsatisfactory results after pubertal growth, while none of the variables was related to size or position of the maxilla. The studies conducted by Baccetti et al^25^ found that orthopedic treatment of Class III malocclusions could be unfavorable when, on initial cephalometric records, patients present a long mandibular ramus (increased posterior facial height), acute skull base angle and inclined mandibular plane.

Fudalej et al^28^ conducted a systematic literature review aiming at assessing the possibility of finding predictors for the results of an interceptive treatment of Class III malocclusions, on the basis of 14 studies selected from a total of 232 publications. It is clear that the review of 14 articles did not present any studies that shared a common model of predictors. On the contrary, there was a significant variety of predictors, although most authors reported huge classification power for the development of a model of predictor (the classification power of predictors varied from 95.6%^29^ to 83.33%^25^). The gonial angle was the variable most frequently identified by the different groups of researchers, it occurred in 36% of the 14 studies analyzed.^27^^,^ 30 ^-^ 34

From this perspective, the aim of this retrospective and longitudinal study was to select a model of cephalometric variables capable of identifying differences in the facial growth of young Class III patients subject to classic orthopedic treatment protocol (RME + FM).

## MATERIAL AND METHODS

The research project that resulted in the development of the present study was approved by the Institutional Review Board of Sacred Heart University under protocol number 117/11. The sample comprised 26 patients, 11 boys and 15 girls, whose initial mean age was of 8 years and 4 months. All patients had maxillary deficiency and/or mandibular prognathism with Class I or Class III malocclusion in mixed dentition. Patients were submitted to RME/FM therapy performed by the same group of clinicians, under standardized procedures. Patients' records were filed at the Center of Surgery and Orthodontics, Bauru, São Paulo/Brazil. In selecting the sample, the following exclusion criteria were considered: previous orthodontic treatment, presence of craniofacial anomalies and significant facial asymmetries.

The diagnosis of Pattern III facial growth was determined on the basis of subjective frontal and profile facial analysis,^5^^,^ 6 ^,^ 35 and confirmed by lateral radiograph of the face.

**Figure 1 -:**
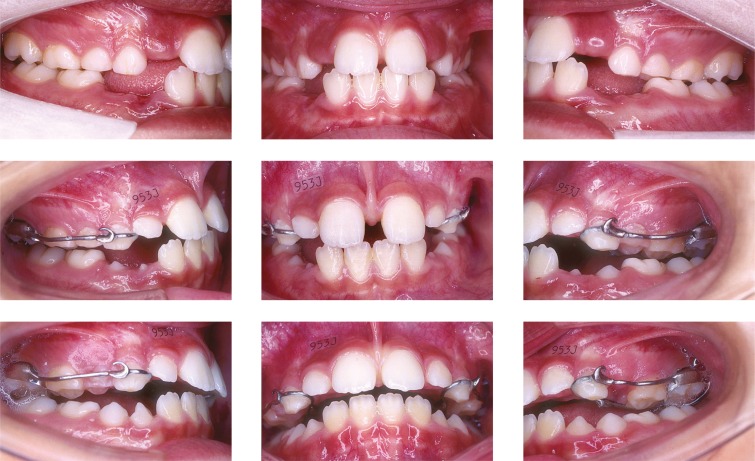
Sequence of orthopedic treatment by means of RME/FM in Class III patients.

The average duration of active orthopedic treatment (RME + FM) was of 6 months. The children began using face mask after expansion was considered clinically evident, for at least 10 hours a day (generally at night). Treatment finished when positive overjet or overcorrection were obtained (Fig 1).

**Figure 2 -:**
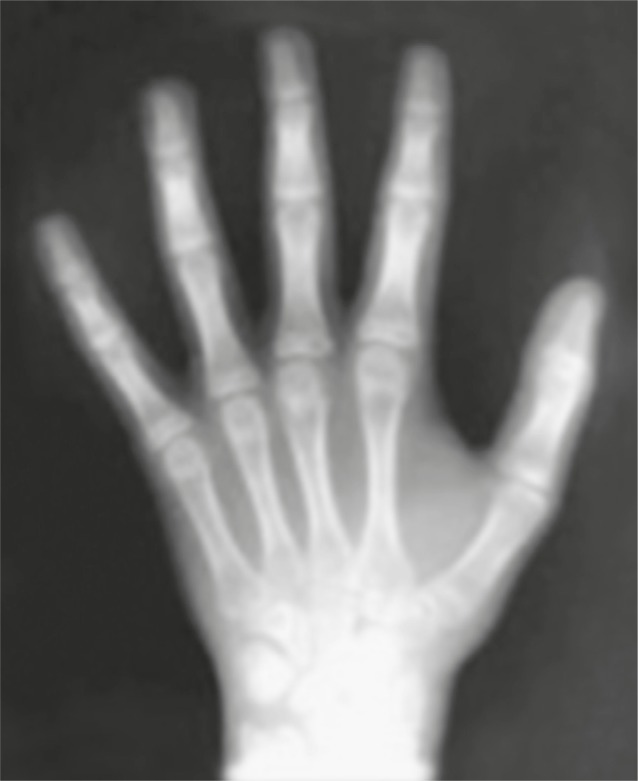
Hand-wrist analysis, IJ stage of the radius in accordance with Hagg and Taranger.36

Patients were monitored after interceptive treatment had been carried out and reassessed after an average period of 6 years and 10 months, without any type of retainer, at the end of pubertal growth (mean age of 15 years) which can be detected by biological indicators such as full pubescence for the boys and two years after menarche for the girls. Whenever necessary, hand-wrist analysis was conducted on the search for IJ stage of the radius, in accordance with maturation indicators and pubertal growth spurt^36^ (Fig 2).

In this phase, all parts comprising the sample were assessed by the same evaluator in order to determine whether interceptive treatment was successful or not, in accordance with the criteria for subjective facial analysis.^5^^,^ 6 ^,^ 35

**Figure 3 -:**
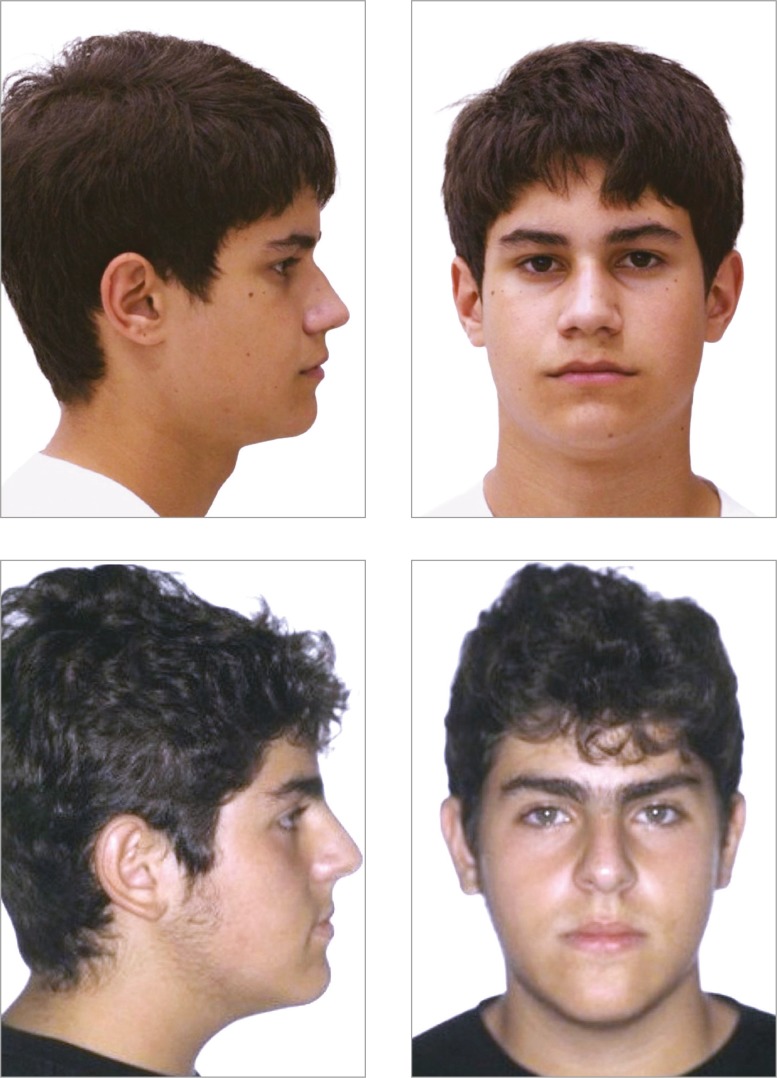
Subjective facial analysis of patients in the successful group.

The successful group comprised 21 patients who, at growth completion, fulfilled the esthetic and functional requirements necessary to be classified as esthetically acceptable, based on a minimum criteria for facial balance, absence of or small asymmetry, and the possibility of having passive labial seal in accordance with parameters previously established.^38^^,^ 39 In addition to technical criteria for subjective facial analysis adopted by the evaluator, patients' self-perception with regard to facial balance is an important factor that influences the decision of those involved in surgical correction. In the successful group, facial esthetics met patients' as well as their guardians' expectations, thus allowing orthodontic treatment to be considered ideal as a primary compensatory measure (Fig 3).

**Figure 4 -:**
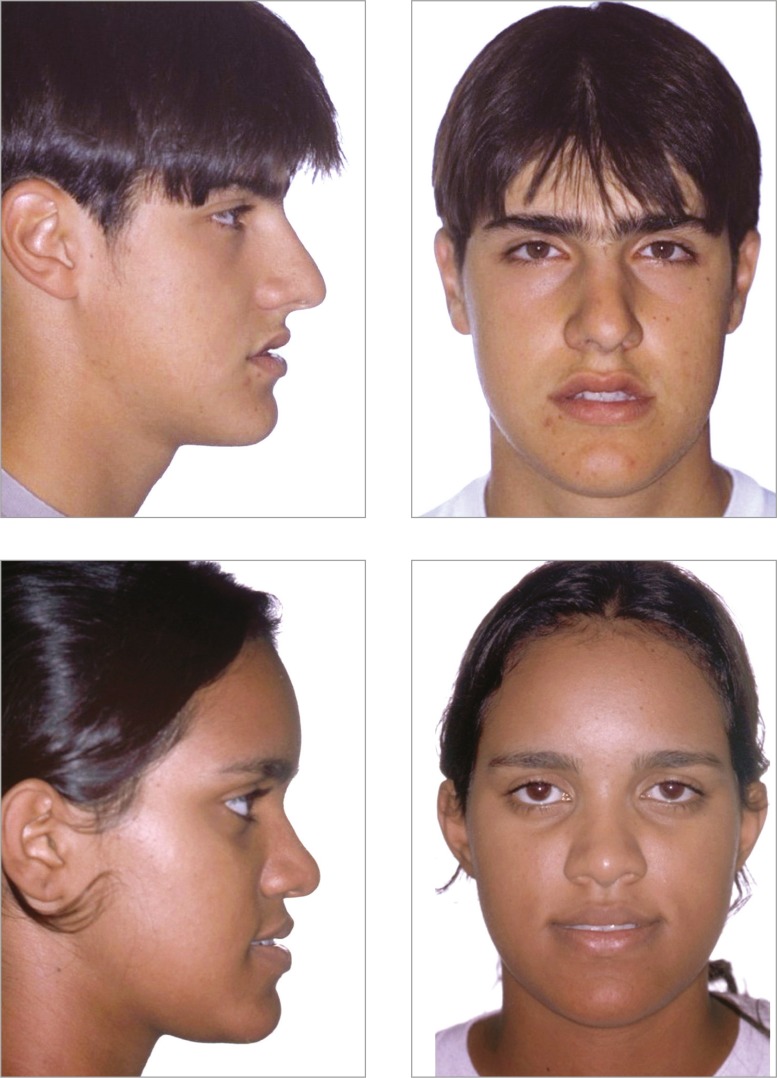
Subjective facial analysis of patients in the unsuccessful group.

Five patients were classified as unsuccessful, being considered as unsatisfactory in terms of facial balance^5^^,^ 35 ^,^ 38 in accordance with the aforementioned patterns, and/or significant malocclusion, which hindered compensatory orthodontic treatment. Therefore, these patients presented needs that could only be eliminated by corrective orthodontic treatment associated with orthognathic surgery (Fig 4).

Lateral radiographs taken at treatment onset were analyzed and adjusted under a magnification factor of 9%. Identification of cephalometric landmarks was based on classic definitions available in the literature.

**Figure 5 -:**
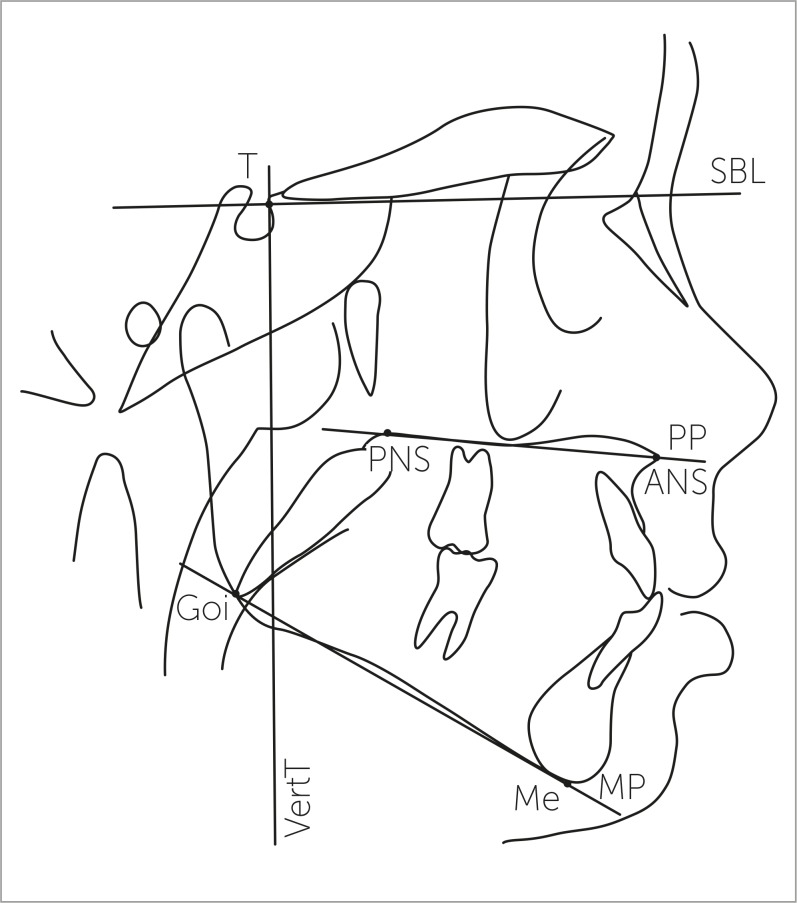
Basicranial reference system. Stable basicranial line (SBL) and vertical line T (VertT). Palatal plane (PP) and mandibular plane (MP).

Cephalometric analysis was based on a basicranial reference system^29^ which was digitized and imported into Radiocef Studio 2 software (Belo Horizonte / MG, Brazil). This system comprised two perpendicular lines - stable basicranial line (SBL) that passes through point T and tangent to the cribriform plate of the ethmoid bone; and vertical line T (VertT) perpendicular to SBL and passing through point T. This point can be easily found in the cephalogram and does not undergo any modification due to growth, as the sella point does.^39^ The stable basicranial line (SBL) used is not remodeled after the patient is 4 to 5 years old.^40^ Two reference lines were also used - palatal plane (PP) that passes through ANS and PNS; and the mandibular plane (MP) that passes through Goi and Me points (Fig 5).

The 18 cephalometric measurements generated ten linear and eight angular measurements, as shown inFigures 6 and7.

**Figure 6 -:**
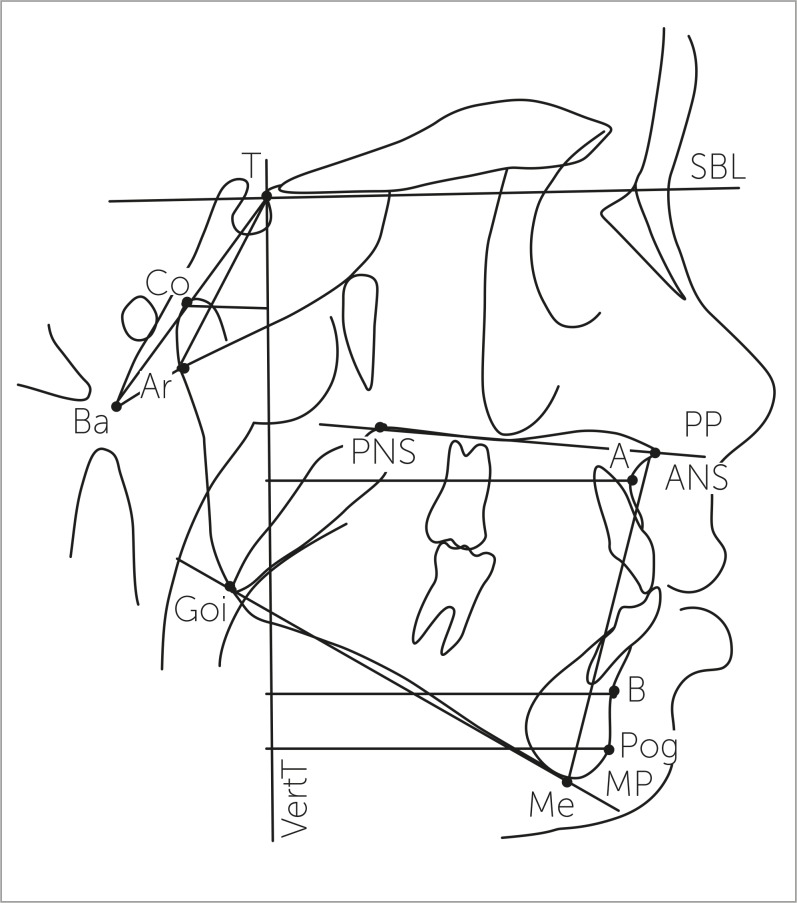
Linear measurements assessing sagittal relationships (A-VertT, B-VertT, Pog-VertT, Co-VertT). Angular measurements for assessing base angulation (Ba.T.SBL, Ar.T.SBL). Linear and angular measurements assessing vertical relationships (PP.SBL, MP.SBL, PP.MP, ALFH).

**Figure 7 -:**
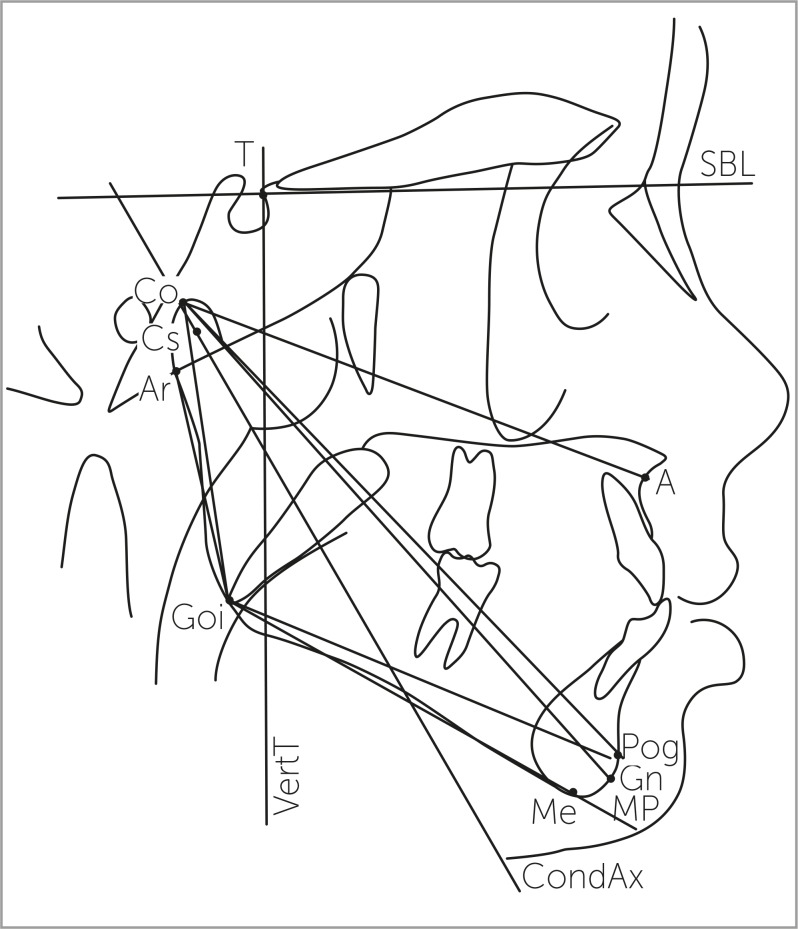
Linear measurements assessing maxillary dimensions (CoA). Linear and angular measurements assessing mandibular dimensions (CoGn, Co-Goi, Goi-Pog, Co-Pog, Ar.Goi.Me) and angular measurements assessing condylar inclination (CondAx.SBL ,CondAx.MP).

The error of the method of all cephalometric measurements was assessed by means of ten radiographs randomly selected. There was a two-week interval between the first and second assessment. T-test was used for intraexaminer systematic error calculation, whereas the random error was calculated by Dahlberg's formula.^41^ The random error ranged from 0.64 to 1.11^o^ for angular measurements and from 0.35 to 1.34 mm for linear measurements.

Discriminant analysis was applied to the cephalometric values of the 26 patients at treatment onset, and the stepwise method was used to identify, among the 18 variables studied, the smallest group that could point out any differences between the two groups determined at growth completion: successful and unsuccessful. In order to attain the best discrimination model, the first phase of analysis consisted in selecting the most important variables in order to divide the groups into successful and unsuccessful.

To verify the normality of groups, Shapiro-Wilk test was employed, with significance level set at 5% (P < 0.05).

## RESULTS

Descriptive statistics for all cephalometric variables in the first observation phase (T_1_), for the total sample and for both groups (successful, n = 21; and unsuccessful n = 5) is shown inTable 1.

**Table 1 -:** Cephalometric variables at T1.

Variable	Total (n = 26)	Success rate (n = 21)	Failure rate (n = 5)
	Mean ± SD	Mean ± SD	Mean ± SD
A-VertT	57.04 ± 6.00	56.30 ± 4.42	60.15 ± 10.59
B-VertT	56.14 ± 7.51	54.69 ± 5.88	62.24 ± 11.07
Co-VertT	15.12 ± 3.03	14.86 ± 3.16	16.18 ± 2.43
Pog-VertT	56.62 ± 8.42	54.79 ± 6.46	64.29 ± 11.95
Ba.T.SBL	52.28 ± 4.63	51.98 ± 4.85	53.53 ± 3.74
Ar.T.SBL	57.28 ± 4.58	57.08 ± 4.86	58.13 ± 3.45
PP.SBL	3.57 ± 2.92	3.15 ± 2.56	5.36 ± 3.93
MP.SBL	22.39 ± 4.81	22.46 ± 4.68	22.08 ± 5.89
PP.MP	24.37 ± 4.52	23.64 ± 4.42	27.44 ± 3.93
ALFH	56.37 ± 5.48	54.93 ± 4.17	62.38 ± 6.68
CoA	75.75 ± 7.37	74.74 ± 5.76	79.95 ± 12.07
CoGn	101.12 ± 10.36	98.64 ± 6.97	111.55 ± 16.12
Co-Goi	48.01 ± 5.38	46.82 ± 3.67	52.99 ± 8.68
Goi-Pog	66.73 ± 6.27	65.60 ± 4.97	71.45 ± 9.38
Co-Pog	99.09 ± 10.15	96.72 ± 7.00	109.01 ± 15.70
Ar.Goi.Me	116.05 ± 5.72	115.24 ± 5.20	119.44 ± 7.17
CondAx.SBL	118.74 ± 4.59	119.01 ± 4.98	117.61 ± 2.46
CondAx.MP	141.13 ± 5.36	141.48 ± 5.57	139.70 ± 4.61

Shapiro-Wilk test showed that all variables were normally distributed, except for: Co-Pog, Co-Goi, Goi-Pog, PP.SBL, CoA and Co-Gn. Since none of these variables remained in the model after discriminant analysis was carried out, the aforementioned normality deviations did not hinder analysis.

**Figure 8 -:**
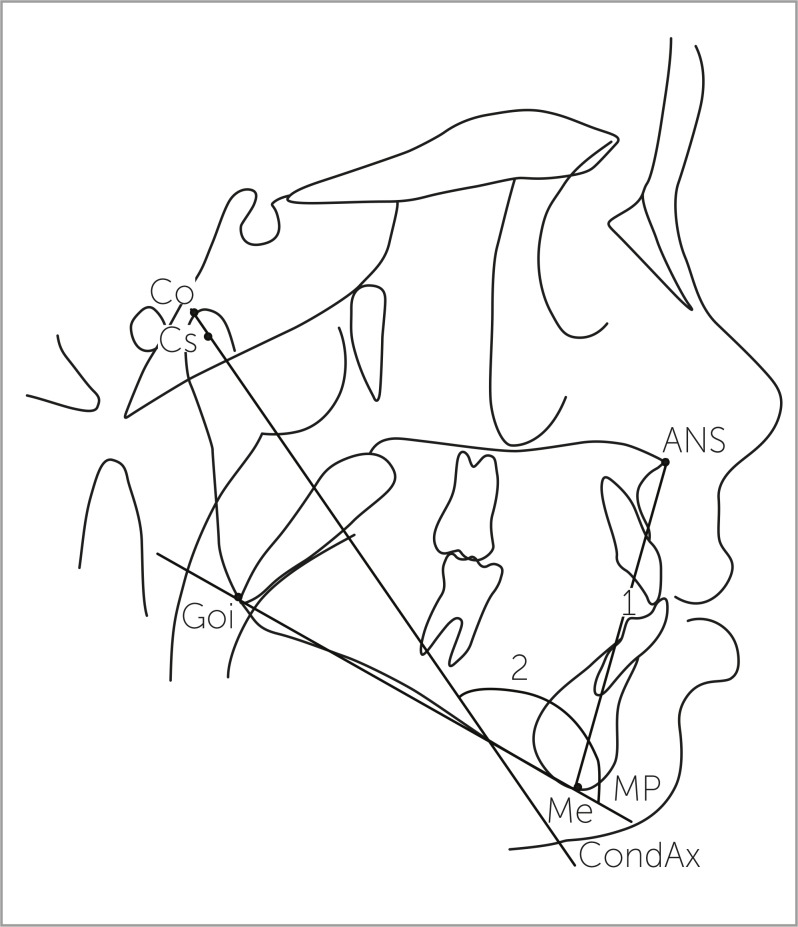
Predictive measurements. 1) ALFH. 2) Angle between the condylar axis and the mandibular plane (CondAx.MP).

Discriminant analysis assesses all variables together, whereas Student's t-test assesses each one separately. Discriminant analysis was performed for all cephalometric variables of the 26 patients at treatment onset (T_1_). In order to attain a better discrimination model that could anticipate the prognosis of an early malocclusion Class III treatment, the stepwise procedure was used to select the variables, using F = 3 in order to keep or remove the variable from the model. When the smallest group of variables was selected, the predictive power (classification power) of the model was tested by means of discriminant analysis. An equation was then developed and applied to all 26 cases, and the total prediction model was calculated. This last procedure provides a prediction model that allows a new patient to be included in each one of the groups. The stepwise procedure identified satisfactory predictors and generated a two-variable model that produced the most efficient division between the two groups. The variables selected were anterior lower facial height (ALFH) and inclination of the condylar axis with the mandibular plane (CondAx.MP), a plane that represents the mandibular body, passing through Goi and Me points (Fig 8 andTable 2). An increased ALFH and a decreased mandibular plane ,in relation to the condylar axis, happening at the same time, signal a predictive capacity towards failure.

**Table 2 -:** Stepwise procedure to select model variables.

Model variables	F = 3	Variables outside the model	F = 3
ALFH	13,801	A-VertT	0,022
CondAx.MP	3,296	B-VertT	0,430
		Co-VertT	0,185
		Pog-VertT	0,672
		Co-Pog	0,019
		Co-Goi	0,257
		Goi-Pog	0,281
		Ba.T.SBL	0,365
		Ar.T.SBL	0.392
		PP SBL	1,211
		MP.SBL	0,159
		PP.MP	1,235
		Ar.Goi.Me	1,951
		CondAx.SBL	0,159
		CoA	0,345
		GoGn	0,000

Value to remove the model F=3.

The classification power of the model for both variables selected was 88.5% (Table 3). Only one in every five patients of each group was not correctly classified. The non-standard discriminant function coefficient of the variables selected, and the calculation of the constant (Table 4) resulted in the equation below, which provides an individual score of attribution that allows each new patient to remain either in the successful or unsuccessful group (equation 1):

**Table 3 -:** Discriminant analysis classification results.

Group	Number of cases	Prediction based on the model			
		Success rate		Failure rate	
		n	%	n	%
Success rate	21	19	90.5	2	9.5
Failure rate	5	1	20.0	4	80.0

Percentage of correctly classified values = 88.5%.

**Table 4 -:** Coefficients produced by discriminant analysis.

Predicting variable	Non-standardized coefficients
ALFH	0.232
CondAx.MP	-0.116
Constant	3.289

Discriminant values (centroid values of groups): Success rate = -0.372; Failure rate = 1.562; Critical value = 0.595.

Discriminant function:

Individual value = 0.232 x ALFH - 0.116 x CondAx.MP + 3.289

The critical value (the value that separates the successful group from the unsuccessful one), i.e., the mean values of the centroid group for both groups, is calculated on the basis of data presented inTable 4, a value of 0.595, which stands for the mean value for the centroid group of both groups. Each new Class III patient, whose value obtained for the discriminant function was below critical value (0.595), shall be treated by means of RME/ FM therapy with prognosis of success. Conversely, patients whose value obtained for the discriminant function was above the critical value shall be treated with prognosis of failure, i.e., they may not have satisfactory results by the end of orthopedic treatment.

## DISCUSSION

Interceptive treatment of Class III malocclusion complies with a protocol most likely to be the most uniform in Orthodontics, and which although upheld by undeniable efficiency,^1^^,^ 3 ^,^ 14 ^,^ 17 ^,^ 24 has some limitations imposed by growth pattern, with progressive and variable losses overtime. Within this context, prognosis depends on the skeletal discrepancy given by Pattern III facial growth,^5^ which causes some concern to the people involved: patient, guardians and the clinician. Other studies have tried to overcome this problem and aim at finding out the discriminant variables; in other words, the characteristics that, once identified in the pre-treatment phase, may contribute to defining a long-term prognosis at the end of growth.

That was the objective of this research. A group of 26 patients submitted to RME/FM therapy had their lateral radiographs of the face assessed before treatment and after a 6-year follow-up, without any type of retainer, by the end of facial growth (mean age of 15 years old). The maturation status was determined by biological indicators and confirmed by hand-wrist radiograph assessment^36^ (Fig 2).

The criteria provided by subjective facial analysis were used to divide the groups at active growth completion, a critical moment for the definition of the therapeutic procedure and, hence, for the success or failure of interceptive therapy. Such criteria were then associated with the identification of predictive measures with significant prognostic power at treatment onset of Class III patients. Facial analysis was used to identify positive and negative facial features and, therefore, suggest a method to treat the occlusion. Success or failure of orthopedic treatment requires growth prediction. No accurate methods employed to predict the future of mandibular growth are yet available.^27^ Our findings specifically aimed at searching for predictability of results yielded by an interceptive treatment protocol of Class III malocclusion.

All 21 patients in the successful group were considered acceptable according to the subjective facial analysis criteria.^5^^,^ 35 ^,^ 37 Despite having a skeletal error that had not been corrected in its essence, these patients' facial pattern displayed a balanced and close-to-normal face. The magnitude of error was not significant enough so that it could be seen in frontal assessment,^38^ in mandibular growth properly related to maxillary growth, and in the signs of excessive mandibular growth - considered as mild. Most of times, it was considered as acceptable, thus suggesting a reduction in profile convexity, which is typical of Classe III patients. Facial asymmetry, whenever present, had no significant progress or impact.

All five patients in the unsuccessful group presented sagittal imbalance, severe enough so that it could be identified even by frontal assessment. Straight or concave profile confirms studies that brought out a direct relationship between profile convexity and unpleasant esthetic appearance,^38^ evident signs of prognathism, mandibular imbalance with regard to size, shape and position, with increased chin-throat length. Morphological signs of maxillary deficiency were clearly identified, transposing the limits of acceptability. The nasogenian fold was evident, zygomatic projection was absent and there were signs of exophthalmia, which damage the medium third of the face, especially if patients' age is considered. Additionally, two out of five patients in the unsuccessful group presented remarkable and severe facial asymmetry.

The results obtained, with 80.7% of the sample considered as successful, confirm the presupposed efficiency of the orthopedic correction protocol (RME/FM) employed as an intervention to treat Class III malocclusion. These data corroborate those found in the literature^19^^,^ 20 ^,^ 24 ^,^ 42 ^,^ 43 which report a similar success rate with regard to this procedure when employed in mixed dentition.

Taking the aforementioned information into account as well as the main purpose of this research, the following question arises: Could patients destined for different results by the end of growth, whether successful or unsuccessful, have a prognosis defined a priori, i.e., before interceptive treatment is performed?

Comparison between the successful and unsuccessful groups, carried out by means of discriminant analysis applied to cephalometric data obtained at treatment onset, revealed two variables with predictive power: ALFH and condyle inclination in relation to the mandibular plane. In practical terms, the smaller number of variables included in the discriminant analysis, the more relevant it will be.^29^^,^ 34 Discriminant analysis was chosen as an efficient technique used to identify the cephalometric variables capable of predicting the results of early orthopedic treatment performed in Class III patients.^25^^,^ 26 ^,^ 29 ^,^ 31 ^,^ 34 Although discriminant analysis was considered as relatively efficient, the authors,^26^^,^ 34 who sought support not only to recommend treatment during growth, but also to wait so that surgical treatment could be carried out, took into account the fact that other factors, such as dimension of the jaws and heredity, should be included in the analysis with the purpose of increasing the ability of prediction.

By means of discriminant analysis, Baccetti et al^25^ identified three cephalometric variables with prognosis ability present at initial cephalometric register: Long mandibular ramus or increased posterior facial height (Co-Goi), greater cranial base angulation (Ba.T.SBL) and increased angle between the mandibular plane and the cranial base(MP.SBL), all of which were related to unfavorable results at active growth completion, with a classification power of variables of 83.33%. Such rate was similar to that found in our research: 88.5%.

Moon et al^34^ reported that patients with a lower value for the gonial angle and a more horizontal skeletal pattern present better prognosis for orthopedic treatment of Class III malocclusion. Additionally, by means of the stepwise procedure employed to select the variables, they obtained the measure between the mandibular plane (Go-Me) and the anteroposterior relationship between the maxilla and the mandible (AB), with higher predictive power, particularly regarding the identification of surgical cases.

Therefore, it seems reasonable to admit that, in both studies,^25^^,^ 34 the results suggest that an increased angle between the mandibular plane and the cranial base (MP.SBL)^25^, as well as lower angle between the mandibular plane and the AB line (AB to Go-Me) define an unsatisfactory prognosis.

In a retrospective study, based on the results of orthopedic treatment (RME/FM) performed in 64 Class III patients, Ghiz, Ngan and Gunel^27^ selected four cephalometric variables with higher predictive power for unsatisfactory treatment results; all variables were related to the mandible: shorter distance from the condyle to the cranial base, shorter length of the ramus, longer mandibular length. The gonial angle was significantly greater in the unsuccessful group, which corroborates the findings of this research (Table 1).

Moreover, changes in facial height can also be considered, since posterior facial height or the length of the ramus (Co-Goi), obtained by Baccetti et al,^25^ increased. Conversely, our results considered increased ALFH as an unfavorable prediction variable at treatment onset. Such fact agrees with previous studies^25^^,^ 28 in which Class III patients with vertical growth pattern are associated with unsatisfactory results.

Franchi et al^29^ accounted Class III patients with greater angle between the palatal plane and mandibular plane in deciduous dentition as an unfavorable prognostic sign, emphasizing the important role vertical standards play.

Thus, in general, it seems that mandibular shape and growth are more important than the initial maxilomandibular sagittal relationship for a long-term prognosis of early Class III malocclusion treatment.^27^^,^ 34

From this broad perspective, comparison between our results and those yielded by other researches^25^^,^ 27 ^,^ 34 presents some limitations, given the interaction between prediction factors, i.e., the presence of variables, at the same time, is what determines the predictive power. In other words, the fact that an increased ALFH and a decreased mandibular plane angle in relation to the condylar axis were present at the same time in our research is what determines the predictive power towards failure (Fig 8).

The limitations of this process of identifying variables capable of expressing prediction and allowing a prognosis are as evident as the possibilities that were raised. The greatest limitation is expressed in the large number of predictors found and in the rare repetition of variables in the predictors models found in the literature. As usual, the search for knowledge should be ongoing. It seems reasonable to consider that including the predictors that occur with higher frequency in the researches carried out, for instance, the gonial angle, the changes in mandibular shape and size as well as the information regarding heredity, in the analysis model, can enhance the prognostic ability.

## CONCLUSION

Based on the results of this retrospective and longitudinal study conducted with young Class III patients who underwent an interceptive orthopedic treatment protocol (RME/FM) and were assessed by the end of facial growth, it is reasonable to conclude that an unfavorable prognosis can be predicted when cephalometric analysis carried out at treatment onset reveals that patients present, at the same time, increased ALFH and decreased value of the angle between the condylar axis and the mandibular plane (CondAx.MP). The outcomes at active growth completion of an interceptive orthopedic treatment performed in Class III patients can be predicted with a hit probability of 88.5%.
